# Electroacupuncture at PC6 or ST36 Influences the Effect of Tacrine on the Motility of Esophagus

**DOI:** 10.1155/2014/263489

**Published:** 2014-04-07

**Authors:** Chi Wang, Xin Chen, Peng-Yan Xie

**Affiliations:** Department of Gastroenterology, Peking University First Hospital, 8 Xishiku Street, Beijing 100034, China

## Abstract

*Aim.* To investigate the mechanisms of gastrointestinal side effects of tacrine, and find treatment methods with electroacupuncture (EA). *Methods*. Twenty-five healthy cats were randomly divided into 5 groups: gastric-distention group (model group), tacrine group (cholinesterase inhibitor), tacrine + sham acupoint group (control group), tacrine + PC6 (neiguan) group, and tacrine + ST36 (zusanli) group, with 5 cats in each group. Saline 2 mL i.p. was given 30 min before gastric distention in model group. Tacrine 5.6 mg/kg i.p. was given 30 minutes before gastric distention in the other groups. Tacrine + sham acupoint group (control group), tacrine + PC6 group, and tacrine + ST36 group received EA at corresponding acupoints during gastric distention. The frequency of TLESRs and LESP were recorded by using a perfused sleeve assembly. *Results*. Compared with the model group, tacrine significantly increased the frequency of gastric distention-induced TLESR (P < 0.05) but did not influence the rate of common cavity during TLESR. Tacrine significantly increased the LESP, which could not remain during gastric distention. EA at PC6 could decrease the frequency of TLESR and maintain the increase of LESP, but EA at ST36 did not have these effects. *Conclusion*. Tacrine can significantly increase the gastric distention-induced transient lower esophageal sphincter relaxations (TLESRs). Electroacupuncture (EA) at PC6 may reverse the above side effect.

## 1. Introduction

Tacrine was the first acetylcholinesterase inhibitor (AChEI) introduced in therapy for the treatment of Alzheimer's disease (AD) [1], which was used to increase the levels of acetylcholine, a neurotransmitter in the brain that was involved in learning and memory [2]. However, tacrine was limited in clinic because of the side effects in gastrointestinal tract such as nausea, vomiting, and regurgitation [3].

Transient lower esophageal sphincter relaxations (TLESRs) are the most important mechanism of gastroesophageal reflux (GER) either in the patients of gastroesophageal reflux disease (GERD) or in normal subjects [4]. Acupuncture has been used to treat functional gastrointestinal disorders in Eastern countries for centuries. It can modulate visceral sensation as well as function through stimulation at selected acupoints along the meridians (channels of acupoints). Our previous studies have suggested that electroacupuncture (EA) at Neiguan (PC6) [5] and Zusanli (ST-36) [6] can inhibit the frequency of TLESR triggered by gastric distention. But the effects of EA on the side effects of tacrine by EA have not been investigated. The aim of our research is to investigate the mechanisms of gastrointestinal side effects of tacrine and evaluate the efficacy of EA at ST-36 or PC6.

## 2. Materials and Methods

### 2.1. Materials

The experiments were performed on 25 adult cats, weighing 3.6 ± 0.2 kg (M/F : 15/10). Cats were provided by the Animal Center of the First Hospital of Peking University. They were kept in individual cages in a controlled environment with a temperature of 22–26°C, 12/12-h light/dark cycles, and fed with standard cat diet. The animals were deprived of food 10 h before each experiment. All procedures were approved by the Committee for Animal Care and Usage for Research and Education of the Peking University. Anesthesia was initially induced with ketamine hydrochloride (30 mg/kg i.m.). Supplementary doses of ketamine hydrochloride (15 mg/kg i.p.) were given whenever necessary to maintain an appropriate depth of anesthesia, as assessed them remained motionless yet still had cornea reflex. They were euthanized with pentobarbital sodium (0.5 mL/kg i.p.) at the end of the protocol.

Tacrine was obtained from Sigma Chemical Co. (St. Louis, MO, USA). Saline was provided from Beijing Shuanghe Chemical Company.

### 2.2. Experimental Groups (Figure 1)

After manometry catheter intubation, the cat accommodated it for 5 min. Then the esophageal manometry was performed for 30 minutes at the baseline and for 60 minutes during gastric distention/gastric distention + EA. The model group only received saline 2 mL i.p. and the other groups received tacrine 5.6 mg/kg i.p. 30 min before gastric distention.

- Gastric-distention group (model group, *n* = 5): received saline 2 mL i.p. 30 minutes before gastric distention.
- Tacrine group (*n* = 5): received tacrine 5.6 mg/kg i.p. 30 minutes before gastric distention.
- Tacrine + sham acupoint (control group, *n* = 5): received tacrine 5.6 mg/kg i.p. 30 minutes before gastric distention and received EA at sham acupoint during gastric distention for 60 minutes.
- Tacrine + PC6 group (*n* = 5): received tacrine 5.6 mg/kg i.p. 30 minutes before gastric distention and received EA at PC6 during gastric distention for 60 minutes.
- Tacrine + ST36 group (*n* = 5): received tacrine 5.6 mg/kg i.p. 30 minutes before gastric distention and received EA at ST36 during gastric distention for 60 minutes.

### 2.3. Recording Methods

The manometry catheter (outer diameter 0.5 cm) consisted of a multilumen silicone tube with five side holes located at 9, 6, 3, 0, and −6 cm from the upper margin of the 6 cm long Dent sleeve sensor (Dentsleeve, Belair, Australia). The catheter was continuously perfused with distilled water by a low compliance pneumohydraulic capillary infusion system (UPS-2020, Holland) at a rate of 0.2 mL/min. The external pressures transducers were connected via an analog/digital converter to a personal computer system. The data were displayed continuously on a monitor and stored on the personal computer system (MMS B.V., The Netherlands). After anesthesia the cat was set in a supine position. A manometry catheter was placed through the mouth into esophagus and positioned so that the sleeve sensor straddled the LES to register LES pressure. The distal side hole was used as a reference point for intragastric pressure. And the upper LES side holes were used to measure esophageal body pressure. A mylohyoid electromyography (MMS B.V., The Netherlands) was used to record swallowing. The pinhead electrode was inserted in the mylohyoid muscle, and the reference electrode was fixed to the interscapular region of the back.

### 2.4. Electroacupuncture

Two acupuncture needles of 0.22 mm in diameter (Suzhou Global Acupuncture Instrument Co., Ltd., Suzhou, China) were inserted perpendicularly at the bilateral Neiguan acupoint (PC6, located 1.5–2.0 cm above the wrist between the ligaments of the flexor carpi radialis and the palmaris longus overlying the median nerve [5]) or Zusanli acupoint (ST36, located at the proximal one-fifth of the craniolateral surface of the rear leg, distal to the head of the tibia in a depression between the muscles of the cranial tibia and the long digital extensor [6]) to a depth of 5 mm. After the needle was inserted, we manipulated with uniform reducing-reinforcing methods to induce the deqi sensations of acupuncturist, such as heavy, tight or even vibration of the needle, and then electrostimulation was introduced. An electrical stimulator (Model LH202H Hans, Beijing Huawei Medical Instrument Co., Ltd., Beijing, China) provided current to the needles. Wave patterns were sparse with dense pulse intervals ranging from 2 to 100 Hz (2/100 Hz), with constant amplitude and current flow (3-4 mA). The duration was 60 min and correct positioning was confirmed by observing slight repetitive paw flexion during stimulation. Control stimulation on a sham acupoint was conducted at the hip, a point away from the traditional meridians and dermatomes.

### 2.5. Gastric Distention

Air was insufflated (at a rate of 15 mL/s) into stomach through a 2.0 mm diameter tube intubated through mouth to stomach. Its depth equals to 5 cm plus the esophageal body length. 30 mL air into stomach every 6 min amount to to 300 mL was insufflated in the 1 h period of gastric distention.

### 2.6. Data Analysis

TLESRs were defined according to established methods [7]. Basal LES pressure was measured at end expiration relative to gastric pressure. The LES pressure during gastric distention was measured for 1 min every 6 min, and an overall mean for each period of the study was calculated. Common cavities were defined as abrupt simultaneous and sustained rises of basal esophageal pressure to intragastric pressure in at least the two lower esophageal body manometry recording sites [8]. Common cavities are considered as markers of gas or liquid reflux from the stomach into the esophagus.

### 2.7. Statistical Analysis

The number of TLESRs was compared using Wilcoxon signed rank test and expressed as median (interquartile range). Basal LESP, postmedicine LESP, and gastric distension LESP were presented as means ± SD and were compared using ANOVA. The rate of common cavity was compared using paired sample *χ* ^2^. SPSS 17.0 was used for statistical analysis, and *P* < 0.05 was considered statistically significant.

## 3. Results

### 3.1. Transient LES Relaxations

#### 3.1.1. Effect of Tacrine on TLESR

**Figure 2:**
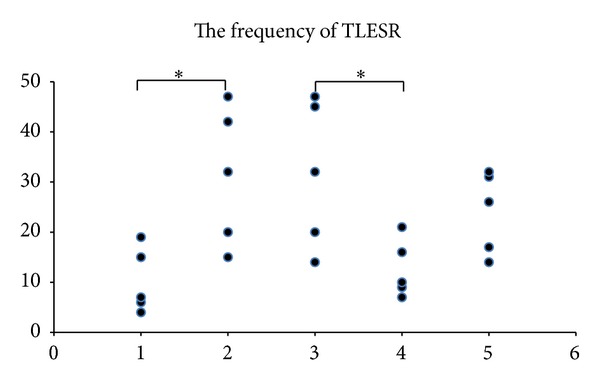
Tacrine significantly increased the frequency of TLESRs compared with model group (*P* < 0.05). EA at PC6 significantly inhibited the frequency of TLESRs than control group (*P* < 0.05). EA at ST36 could not change the frequency of TLESRs compared with control group (*P* = 0.388). *P < 0.05: (1) gastric distention group (model group); (2) tacrine group; (3) tacrine + non-acupoint (control group); (4) tacrine + PC6; and (5) tacrine + ST36.

Tacrine [32/hour (range: 15–47)] significantly increased the frequency of gastric distention-induced TLESRs compared with model group [7/hour (range: 4–19)], *P* < 0.05. (Figure 2).

#### 3.1.2. Effect of Tacrine + EA on TLESR

After the use of tacrine, EA at PC6 [10/hour (range: 7–21)], the frequency of gastric distention-induced TLESR, was significantly inhibited than that of the control group [32/hour (range: 14–47)], *P* < 0.05. (This result was consistent with our former research [5].) EA at ST36 [26/hour (range: 14–32)] could not change the frequency of gastric distention-induced TLESR compared with the control group [32/hour (range: 14–47)], *P* = 0.388. (Figure 2)

### 3.2. Effect of Tacrine on Common Cavity

Tacrine had no effect on common cavity rate during TLESR. In model group, a total of 51 TLESRs were induced by gastric distention, among them there were 37 associated with common cavity, and the ratio was 72.5%. In tacrine group, there were 156 gastric distention-induced TLESRs in all, from which 98 were associated with common cavity, and its ratio was 62.8%. (72.5% versus 62.8%, *P* = 0.238).

### 3.3. Lower Esophageal Sphincter Pressure (LESP)

There were three parts of LESP in the whole protocol: baseline LESP, postmedicine (tacrine/saline) LESP, and gastric distention LESP.

#### 3.3.1. Effect of Tacrine on LESP

**Figure 3:**
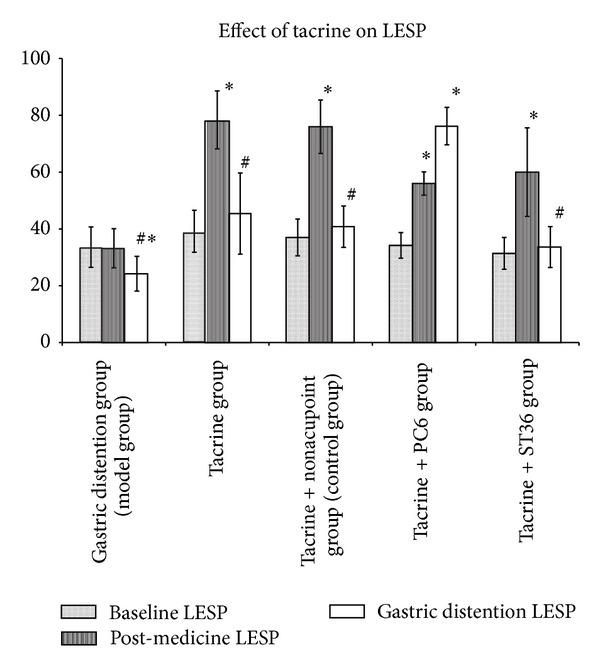
(a) In model group, there was no significant difference between baseline LESP and postmedicine LESP. The gastric distention LESP was significantly lower than baseline LESP and postmedicine LESP. In tacrine group, the postmedicine LESP was significantly higher than baseline LESP and gastric distention LESP; there was no significant difference between baseline LESP and gastric distention LESP. (b) In control group and tacrine + ST36 group, the postmedicine LESP was significantly higher than baseline LESP. And the gastric distention LESP was falling to the baseline level. In tacrine + PC6 group, the postmedicine LESP was significantly higher than baseline LESP. And the gastric distention LESP did not decline and even had ascending trend (*P* = 0.086). *P < 0.05 versus baseline LESP. ^#^ *P* < 0.05 versus postmedicine LESP.

In model group, there was no significant difference between baseline LESP and postmedicine LESP (33.6 ± 7.1 mmHg versus 33.2 ± 6.9 mmHg, *P* = 0.374). The gastric distention LESP was significantly lower than baseline LESP (24.2 ± 6.1 mmHg versus 33.6 ± 7.1 mmHg,* P *< 0.05) and postmedicine LESP (24.2 ± 6.1 mmHg versus 33.2 ± 6.9 mmHg, *P* < 0.05). In tacrine group, the postmedicine LESP (78.4 ± 10.2 mmHg) was significantly higher than baseline LESP (39.2 ± 7.4 mmHg) and gastric distention LESP (45.4 ± 14.3 mmHg) *P* < 0.05, and there was no significant difference between baseline LESP and gastric distention LESP (39.2 ± 7.4 mmHg versus 45.4 ± 14.3 mmHg, *P* = 0.651) (Table 1, Figure 3).

#### 3.3.2. Effect of Tacrine + EA on LESP

In control group and tacrine + ST36 group, the postmedicine LESP was significantly higher than baseline LESP (*P* < 0.05) and the gastric distention LESP was falling to the baseline level (gastric distention LESP versus postmedicine LESP, *P* < 0.05 and gastric distention LESP versus baseline LESP, *P* > 0.05) (Figure 3). In tacrine + PC6 group, the postmedicine LESP was significantly higher than that of baseline (56.0 ± 4.1 mmHg versus 34.2 ± 4.5 mmHg, *P* < 0.05) and the gastric distention LESP did not decline and even had ascending trend (gastric distention LESP 76.2 ± 6.6 mmHg versus postmedicine LESP 56.0 ± 4.1 mmHg, *P* = 0.086) (Table 1, Figure 3).

## 4. Discussion

### 4.1. Effects of Tacrine

Tacrine is the first acetylcholinesterase inhibitor (AChEI) introduced in therapy for the treatment of Alzheimer's disease (AD) [1], but its clinical application is limited because of the side effects in gastrointestinal tract such as nausea, vomiting, and regurgitation [3]. In this study, we investigated the mechanisms of gastrointestinal side effects induced by tacrine. Acetylcholine is not only a neurotransmitter involved in learning and memory [2], but also an important neurotransmitter in esophageal-gastric motility, especially in regulating TLESR. As we know, atropine, an anticholinergic agent with central and peripheral actions, can inhibit the frequency of TLESR and some selective peripheral cholinergic blockade did not reduce it, so a central cholinergic pathway may be involved in regulation of TLESR. Tacrine is a kind of acetylcholinesterase inhibitor (AChEI), which works through increasing the level of acetylcholine in brain.

In this study, the relationship between tacrine and esophageal motility was investigated. We firstly observed that tacrine significantly increased the frequency of gastric distention-induced TLESR compared with model group, but tacrine did not change the common cavity rate during TLESR. TLESR was the most important mechanism of gastroesophageal reflux [4]. The effect of tacrine to increase the frequency of gastric distention-induced TLESRs may be the most important reason for nausea, vomiting, and regurgitation.

At the same time, tacrine significantly increased the LESP compared with baseline LESP. Although tacrine may increase LESP, this increase could not maintain during gastric distention, so it could not inhibit the gastroesophageal reflux during gastric distension.

### 4.2. Effects of Tacrine + EA

Acupuncture has been used to treat functional gastrointestinal disorders in Eastern countries for centuries. A large amount of clinical evidence supports the effectiveness of acupuncture for treating functional disorders of the gastrointestinal tract, and the most commonly used acupoints in treating gastrointestinal symptoms are Neiguan (PC6) and Zusanli (ST-36). Based on another experiment in our research group, electroacupuncture (EA) at PC6 [5] and ST-36 [6] could inhibit the frequency of TLESR triggered by gastric distention.

Our previous research showed that after using tacrine and EA at PC6, the frequency of gastric distention-induced TLESR was significantly decreased compared with control group [5]. In this study, it was further demonstrated that EA at PC6 could also inhibit the decrease of LESP due to gastric distention and even had ascending trend (*P* = 0.086). In the present study, EA at PC6 may increase the LESP after increase the level of acetylcholine in brain and peripheral tissue, however, in our previous research only EA at PC6 had no effect on LESP [5]. It indicated that EA at PC6 was an attractive therapeutic option to treat the gastrointestinal side effects of tacrine. Further study needed to be done about the mechanism about the EA at PC6 treating the gastrointestinal side effects due to tacrine.

Previous study also showed that EA at ST36 increased LESP and decreased the frequency of TLESR [6]. While in this study, after the level of acetylcholine was increased, EA at ST36 did not have these effects. The results suggested that the effects of EA at ST36 were through acetylcholine pathway.

### 4.3. Deqi Sensation in Animal Experiment

The term of “Deqi” was first found in “Huang Di Neijing” [9]. It plays an important role in the process of acupuncture and it is closely related to the treatment efficacy. Deqi does not only refer to needling sensations, but also involves the changes of qi induced by needle insertion into the acupoint. Some research has found that EA at acupoint (deep needling) induces significant stronger deqi sensation than EA at acupoint (subcutaneous needling) or at nonacupoint [10]. In our research, we use deep needling at PC6 or ST36. After inserting the needle, we manipulated with uniform reducing-reinforcing methods to induce the deqi sensations of acupuncturist, such as heavy, tight or even vibration of the needle, and then electrostimulation was introduced. By this method we may induce deqi sensation and obtain better results. But this still needs further research.

## 5. Conclusion

Tacrine may have some effects on esophageal motility, such as increasing the frequency of gastric distention-induced TLESR and elevating LESP. And this LESP elevation may not persist during gastric distention. EA at PC6 can decrease the frequency of TLESR and maintain the increase of LESP, but EA at ST36 does not have these effects.

## Figures and Tables

**Figure 1:**
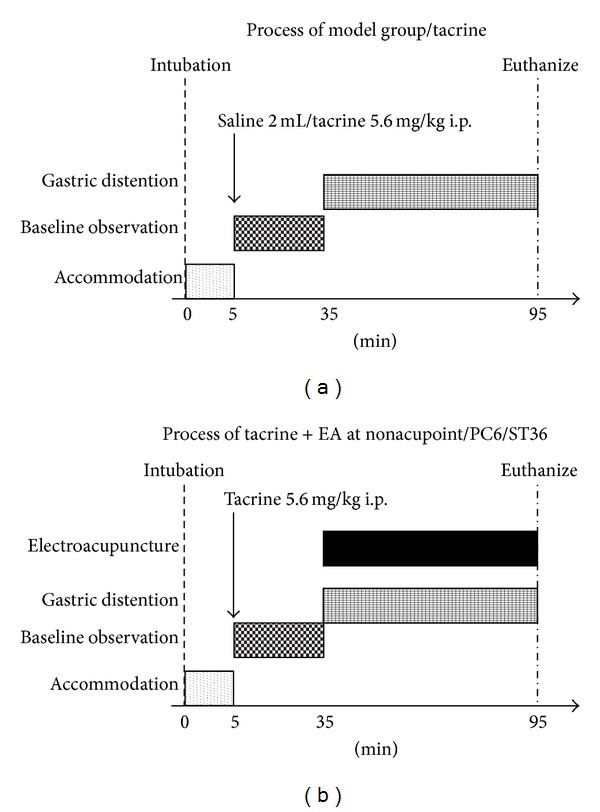
After manometry catheter intubation, the cat accommodated it for 5 min. Then the esophageal manometry was performed for 30 min at the baseline and for 60 min during gastric distention or gastric distention + EA. The model group only received saline 2 mL i.p. and the other groups received tacrine 5.6 mg/kg i.p., 30 min before gastric distention.

**Table 1:** The effects of tacrine and tacrine + EA on LESP.

Group	Baseline LESP	Postmedicine LESP	Gastric distention/gastric distention + EA LESP
Model group	33.6 ± 7.1 mmHg	33.2 ± 6.9 mmHg	24.2 ± 6.1 mmHg^∗#^
Tacrine group	39.2 ± 7.4 mmHg	78.4 ± 10.2 mmHg*	45.4 ± 14.3 mmHg^#^
Tacrine + nonacupoint	37.0 ± 6.5 mmHg	76.0 ± 9.4 mmHg*	40.8 ± 7.3 mmHg^#^
Tacrine + PC6	34.2 ± 4.5 mmHg	56.0 ± 4.1 mmHg*	76.2 ± 6.6 mmHg*
Tacrine + ST 36	31.4 ± 5.6 mmHg	60.0 ± 15.6 mmHg*	33.6 ± 7.2 mmHg^#^

*P < 0.05 versus baseline LESP. ^#^ *P* < 0.05 versus postmedicine LESP.
